# Dietary restriction in senolysis and prevention and treatment of disease

**DOI:** 10.1080/10408398.2022.2153355

**Published:** 2022-12-09

**Authors:** Sepideh Aminzadeh-Gohari, Barbara Kofler, Chiara Herzog

**Affiliations:** a Research Program for Receptor Biochemistry and Tumor Metabollism, Department of Pediatrics, University Hospital of the Paracelsus Medical University, Salzburg, Austria; b European Translational Oncology Prevention and Screening Institute, Universität Innsbruck, Innsbruck, Austria; c Research Institute for Biomedical Ageing, Universität Innsbruck, Innsbruck, Austria

**Keywords:** aging, dietary restriction, disease prevention, epigenetics, senescence, senolysis, senolytics

## Abstract

Aging represents a key risk factor for a plethora of diseases. Targeting detrimental processes which occur during aging, especially before onset of age-related disease, could provide drastic improvements in healthspan. There is increasing evidence that dietary restriction (DR), including caloric restriction, fasting, or fasting-mimicking diets, extend both lifespan and healthspan. This has sparked interest in the use of dietary regimens as a non-pharmacological means to slow aging and prevent disease. Here, we review the current evidence on the molecular mechanisms underlying DR-induced health improvements, including removal of senescent cells, metabolic reprogramming, and epigenetic rejuvenation.

## Less is more – when dietary restriction aids health

We are what we eat: nutrition plays a key role in human health. As early as 1935, McCay et al. demonstrated that diet influences lifespan, when they showed that rats fed a calorie-restricted diet (CR) exhibited a longer mean and maximum lifespan than their *ad libitum*-fed counterparts (McCay, Crowell, and Maynard 1935; McCay et al. 1939). A plethora of preclinical and clinical studies in organisms ranging from yeasts to rodents to humans have since assessed the health impacts of ‘restrictive diets’ (hereafter termed dietary restriction, DR; see Box 1), including CR, intermittent fasting (IF), alternate day fasting (ADF), time-restricted feeding (TRF), or ketogenic diets (KD). CR comprises a reduction of food intake without incurring malnutrition; IF, ADF and TRF consist of alternating cycles of fasting and eating; and KD restricts carbohydrate intake.

There is now ample evidence that DR may improve cognitive function in aging, reduce the accumulation of aged (senescent) cells in blood and tissues, and ultimately increase lifespan or perhaps more importantly, healthspan (i.e., the time prior to the onset of age-associated disease) (Luo et al. 2010; Kauffman et al. 2010; Pifferi et al. 2018; Mattison et al. 2012; Colman et al. 2009) (Tables 1 and 2).

In this review, we discuss the link between DR, cellular senescence, metabolism, and epigenetics. We summarize current knowledge of the molecular mechanisms of DR regimens and how elimination of senescent cells, i.e., senolysis, may contribute to disease relief in old age, with a focus on cancer as an age-related disease.

## Senescence as a cellular basis for age-associated disease

Human cells are known to exhibit a limited capacity to divide (‘Hayflick limit’) (Hayflick 1965; Hayflick and Moorhead 1961) after which they enter a stage called replicative senescence. This phenomenon was first described in cell culture but has since been found in vivo (Muñoz-Espín and Serrano 2014). Progressive telomere attrition during increasing numbers of cell divisions activates the DNA damage response, triggering cell cycle arrest (McHugh and Gil 2018). Senescence can also be elicited by cellular stressors, including hypoxia, mitochondrial and lysosomal dysfunction, oxidative stress, DNA damage, or oncogene activation (Muñoz-Espín and Serrano 2014; Sharpless and Sherr 2015). Senescent cells are stably arrested in cell cycle but show high metabolic and transcriptional activity (Young and Narita 2009; Sikora, Bielak-Zmijewska, and Mosieniak 2019; Sabbatinelli et al. 2019).

The accumulation of senescent cells in tissues occurs in response to many different endogenous triggers, such as age-related dysfunction of cell-mediated clearance, mitochondrial signaling, epigenome and chromatin organization, cytokines and chemokines, (Yousefzadeh et al. 2021; Ovadya et al. 2018), or exogenous triggers such as obesity (Wang et al. 2009; Shirakawa et al. 2016; Palmer et al. 2019) and cigarette smoking (Nyunoya et al. 2006; Paschalaki, Starke, Mercado, et al. 2013; Paschalaki, Starke, Hu, et al. 2013; Baskara et al. 2020). A well-functioning immune system is critical for healthy aging, and senescent cells may impair healthy aging and cause disease via the immune axis (Hashimoto et al. 2019). In line with the wider field of aging research, senescent cell burden has become relevant over the last decade due to an increase in human lifespan - prior to this, the majority of individuals did not live long enough to accumulate a detrimental critical mass of senescent cells.

Senescence is traditionally thought of as a tumor-suppressive mechanism, rendering cells that have encountered genotoxic stresses as non-proliferative (Sikora, Bielak-Zmijewska, and Mosieniak 2019). Moreover, cellular senescence has vital roles during wound healing and development, both of which are two molecular processes that must be tightly spatially and temporally coordinated. Senescence is a programmed but self-limiting response during optimal wound repair, ensuring generation of “appropriate” amounts of fibrosis and remodeling of the extracellular matrix (Jun and Lau 2010a), for instance via release of platelet-derived growth factor AA (Demaria et al. 2014). Notably, dysregulation of the timing or amount of senescence may cause loss of tissue function and impaired wound healing. Programmed senescence also plays a vital role in morphogenesis during vertebrate development, modulating tissue remodeling and patterning (Muñoz-Espín et al. 2013; Storer et al. 2013; Davaapil, Brockes, and Yun 2016; Villiard et al. 2017). Yet, the role of senescent cells is not exclusively beneficial, particularly with increasing age. While senescent cells can protect from malignancies short-term (Halazonetis, Gorgoulis, and Bartek 2008) and aid in tissue remodeling and regeneration (Jun and Lau 2010b; Krizhanovsky et al. 2008), their prolonged presence or accumulation may contribute to impaired wound healing, organ dysfunction, a shortened lifespan, and “inflammaging”. Inflammaging is characterized by persistent low-grade sterile inflammation, impaired immunosurveillance or immune exhaustion (Campisi et al. 2019), factors that are known to contribute to cancer development (Kasler and Verdin 2021; Ferrucci and Fabbri 2018; Leonardi et al. 2018). In general, aging is the single biggest risk factor for cancer alongside constituting a risk for factor many other diseases (Niccoli and Patridge Curr Biol 2012), and approximately 20% of cancers are thought to be caused by chronic inflammation (Mantovani et al. 2008; Balkwill and Mantovani 2012). Inflammaging and the senescence-associated secretory phenotype (SASP) of senescent cells, which includes secretion of pro-inflammatory cytokines, immune modulators, growth factors, and proteases into the tissue microenvironment, have been shown to elicit bystander senescence and cause stem cell exhaustion (Acosta et al. 2013; Hubackova et al. 2012; Chang et al. 2016; Molofsky et al. 2006), contribute to expansion of preneoplastic cells (Krtolica et al. 2001; Ohanna et al. 2011; Lasry and Ben-Neriah 2015; Coppé et al. 2008; Gonzalez-Meljem et al. 2017), drive tumorigenesis (Gonzalez-Meljem et al. 2018; Ruhland et al. 2016), and increase cancer invasiveness (Ghosh et al. 2020; Benítez et al. 2021). Krtolica et al. have termed this duality of senescent cell function – both tumor-suppressive and tumor-driving – as an example of evolutionary antagonistic pleiotropy (Krtolica et al. 2001). The diverse roles of senescent cells and SASP in tumori-genesis were recently summarized by Wang et al. (Wang, Lankhorst, et al. 2022).

Age-related diseases, potentially caused by accumulation of senescent cells, may exacerbate age-related dysfunction, and accrete further disease. For example, recently it was shown that Alzheimer’s disease led to impaired mitochondrial function and increased inflammation and resulted in decreased cardiac contractility in mice (Murphy et al. 2022). Controlling senescence-associated inflammation and clearance of senescent cells have been proposed as means for both prevention and treatment of cancer and other age-related diseases (Lasry and Ben-Neriah 2015; Kirkland and Tchkonia 2017; Wang, Lankhorst, et al. 2022). Depletion of senescent cells has been shown to exert anti-aging effects on stem cells in mice (Chang et al. 2016; Xu et al. 2015). Furthermore, depletion of genes or proteins involved in cell cycle regulation and senescence, such as *CDKN2A* (thereafter referred to as *p16INK4a* [gene] or p16INK4a [protein]), can rejuvenate aged muscle stem cells (Sousa-Victor et al. 2014; S. R. Kim et al. 2021), indicating a potential for reversal of senescence features. Consistent with this, removal of senescent cells has been shown to increase lifespan and delay onset of age-associated diseases, including cancer, in various mouse models (Baker et al. 2011; Baker et al. 2016; Demaria et al. 2014). DR has been proposed to achieve its beneficial effects on lifespan, health, and cancer reduction, at least in part, by reducing or removing senescent cells (Fontana, Nehme, and Demaria 2018; Longo and Cortellino 2020; Cheng et al. 2014) and may thus provide an attractive strategy for non-pharmacological disease prevention.

Pharmacological agents targeting senescent cells in the context of human disease are also of increasing interest. Conceptually, there is a distinction between agents that target senescence via removal of senescent cells (*senolytics*) or via modulation of the senescence-associated phenotype (*senomorphics*). Common senolytics include dasatinib (D) and quercetin (Q), both of which are BCL-2 family inhibitors, and fisetin, a naturally occurring flavonoid (Yousefzadeh et al. 2018). Senomorphics include agents such as rapamycin, metformin, or resveratrol, a natural compound found in red grape skins and other food sources (Zhang, Pitcher, Prahalad, et al. 2022). A key difference in senolytics versus senomorphics is that senolytics could be used for intermittent dosing regimens as they remove the presumed underlying cause (accumulation of senescent cells), whereas senomorphics do not directly remove the underlying senescent cell burden and their effect is likely dependent on continuous presence of the senomorphic agent. There is a number of ongoing human studies investigating senolytics and senomorphics (Zhang, Pitcher, Yousefzadeh, et al. 2022; Gasek et al. 2021), for instance in the context of kidney disease and fibrosis (Clinical Trials NCT02848131 [D or Q]; NCT02874989 [D + Q]; NCT03325322 [fisetin]), frailty (NCT03675724 [fisetin]), Alzheimer’s disease (NCT04785300 [D or Q], NCT04685590 [D + Q]), osteoarthritis (NCT05276895 - planned [D or fisetin]), and to alleviate dysfunction and decrease complications of COVID-19 (NCT04771611, NCT04537299, and NCT04476953 [all fisetin]). The above list of trials is non-exhaustive, and a more detailed description of ongoing trials and agents is provided by Zhang, Pitcher, Yousefzadeh, et al. (2022) and Gasek et al. (2021).

In Table 3, we have compiled information on a number of recent murine studies addressing the role of senescent cells and their elimination in health, longevity, and disease. A comprehensive list of additional preclinical studies utilizing specifically pharmacological/small molecule agents to target senescent cells is provided by two recent publications by Zhang, Pitcher, Yousefzadeh, et al. 2022 and Zhang, Pitcher, Prahalad, et al. 2022).

## Epigenetics of aging

Aging is associated with distinct epigenetic changes, such as alterations in DNA methylation (DNAme), histone modification, and chromatin remodeling. Age-dependent altered methylation of cytosine (5-methyl-C) in the CpG context, which includes a global decrease in methylation (hypomethylation) in repetitive genomic regions and interspersed elements (Bollati et al. 2009; Jintaridth and Mutirangura 2010) and increased methylation (hypermethylation) in promoter regions, is one of the most striking hallmarks of aging and may be a useful biomarker of aging and healthspan (Horvath and Raj 2018). Age-associated hypermethylated promoter regions are frequently found at tumor suppressor genes (Siegmund et al. 2007) and genes involved in cellular fate and differentiation (e.g., polycomb group target genes, PCGTs) (Maegawa et al. 2010; Teschendorff et al. 2010; Rakyan et al. 2010). Deregulation of epigenetic control with age is associated with progressive diseases such as cancer and diabetes (Egger et al. 2004).

While causality is not yet proved, several lines of evidence point to a significant contribution of epigenetic alterations to the aging process: 1) methylation alterations cause chromosomal instability (Esteller and Herman 2002) and contribute to gene expression alterations, transcriptional ‘noise’, and increased transcriptional cell-to-cell variability associated with age and/or cancer (Hernando-Herraez et al. 2019); 2) the aging process in offspring can be modulated by epigenetic alterations accumulated in a parent (e.g., the offspring of younger paternal mice develop aging phenotypes later and live longer than the offspring of aged paternal mice (Xie et al. 2018)); and 3) during cellular reprogramming, amelioration of age-associated phenotypes is observed, highlighting a role for epigenetic remodeling as a driver for aging (Ocampo et al. 2016). Global hypomethylation has been suggested to result in chromatin instability (Esteller and Herman 2002) and has been correlated with frailty in elderly patients (Bellizzi et al. 2012) and increased tumor incidence in mice (Howard et al. 2008). Lastly, the finding that cancers exhibit methylation changes in genes associated with age and stem cell signatures (e.g., PCGTs) (Widschwendter et al. 2007) has led to the progenitor model: epigenetic deregulation, inter alia through age or senescence, can render cells more likely to develop cancer.

Although epigenetic alterations are associated with, and possibly causal for, aging and cancer, DNAme exhibits remarkable plasticity. Age- and senescence-associated DNAme changes can be reversed in vitro by reprogramming to pluripotent (Koch et al. 2013; Frobel et al. 2014; Horvath 2013; Weidner et al. 2014) or multipotent stem cells (Sheng et al. 2018), and DNAme rejuvenation in vivo has been achieved by environmental enrichment or partial reprogramming in mice (Browder et al. 2022) and dietary interventions in humans (Gensous et al. 2020).

Above, we primarily discuss age-associated changes relating to DNAme, yet many other forms of epigenetic changes with age have been described. For instance, aging changes histone levels and nucleosome occupancy, distribution and utilization of histone variants, and histone modification (e.g. acetylation and methylation) (Yi and Kim 2020; Pal and Tyler 2016). An altered chromatin structure and loss of heterochromatin with aging can moreover lead to activation of transposable elements (Pal and Tyler 2016; Andrenacci, Cavaliere, and Lattanzi 2020; Cecco et al. 2013). A detailed discussion of all age-associated epigenetic changes is beyond the scope of the current review, but excellent articles (including, but not limited to, the abovementioned) are available as further reading (Zhang et al. 2020; Wang et al. 2022).

## Molecular mechanisms of DR and healthspan extension

DR without malnourishment can produce beneficial metabolic effects such as reduction of plasma glucose levels and induction of ketosis. The mechanisms by which DR delays or inhibits aging are not fully understood, but DR appears to simultaneously influence multiple cellular pathways. Growing evidence indicates that ketone bodies, especially β-hydroxybutyrate (βHB) as the predominant ketone body in blood, are major mediators of the benefits of CR, fasting, and KD, and are involved in several “anti-aging” mechanisms. In the following section, we highlight potential molecular mechanisms underlying the anti-senescent effects of DR and provide further details from preclinical and clinical studies in Tables 1 and 2.

### Lysosomes and autophagy

Lysosomes are specialized organelles involved in the breakdown of macromolecules via the process of autophagy and undergo prominent senescence-related changes (White, Mehnert, and Chan 2015; Liu et al. 2017). During aging and/or tumorigenesis, insufficient or failed autophagy drives mitochondrial dysfunction and enhanced oxidative stress, DNA damage and genomic instability (White, Mehnert, and Chan 2015; Liu et al. 2017; Rajendran et al. 2019). Inadequate levels of autophagy result in long-term persistence of senescent cells (Rajendran et al. 2019). Stimulation of autophagy in Drosophila melanogaster and mice has been shown to extend lifespan, while its inhibition shortened lifespan (Aman et al. 2021). Several murine studies indicate that DR boosts the autophago-lysosome pathway in the liver, pancreas, muscle, myocardium, brain, lung, and spinal cord, leading to an attenuation of age-associated diseases (Martinez-Lopez et al. 2017; Liu et al. 2017; Yu et al. 2020; Wang et al. 2018; McCarty, DiNicolantonio, and O’Keefe 2015; Yuan et al. 2021; Mu et al. 2021), which may be mediated by activation of AMP-activated kinase (AMPK) and subsequent downregulation of mammalian target of rapamycin (mTOR), elevation of free fatty acids, and activation of sirtuin 1, amongst other potential pathways (Martinez-Lopez et al. 2017; Liu et al. 2017; Yu et al. 2020; Wang et al. 2018; McCarty, DiNicolantonio, and O’Keefe 2015; Yuan et al. 2021; Galluzzi et al. 2014; Mu et al. 2021). DR moreover reduces the cellular access to nutrients such as glucose and amino acids in the extracellular fluids, which triggers autophagy (Galluzzi et al. 2014). βHB is a key factor mediating DR-induced autophagy during glucose deprivation, as it stimulates the autophagic flux and prevents autophagosome accumulation (Camberos-Luna et al. 2016; Torres-Esquivel et al. 2020). Recent studies suggest that circadian rhythms involved in autophagy may be critical for the beneficial effects of DR (Ulgherait et al. 2021; Jamshed et al. 2019), but more research is required to understand the underlying molecular mechanisms.

### Mitochondrial dysfunction and reactive oxygen species

Senescent cells accumulate damaged mitochondria (Park et al. 2018; Vernier and Giguere 2021). Peroxisome proliferator-activated receptors (PPARs), in coordination with coactivators such as PPARγ coactivator 1α (PGC-1α), regulate mitochondrial function and biogenesis (Duszka et al. 2020), and aberrant PPAR/PGC-1α activity is considered to be the main reason for impaired mitochondrial bioenergetics and function in senescent cells (Vernier and Giguere 2021; Duszka et al. 2020). Mitochondrial dysfunction induces the generation of reactive oxygen species (ROS) and can lead to oxidative stress, contributing to aging as well as a variety of pathologies such as diabetes, cancer, and cardiovascular and neurodegenerative disease (Duszka et al. 2020). While free radicals may somewhat contribute to disease, the free radical theory of aging (later oxidative stress theory of aging (Lin and Beal 2003)) has been cast into doubt by results from several animal studies: overexpression of antioxidant genes has been found to have little influence on lifespan (Pérez et al. 2009). One exception to this were findings in an animal model with overexpression of catalase targeted to mitochondria (mCAT) (Schriner et al. 2005), which suggested that specific targeting of antioxidants to mitochondria may be beneficial for healthspan. Further work to dissect the role mitochondria-targeted interventions in the framework of a new “mitochondrial free radical theory” in aging (Dai et al. 2014) is required.

βHB treatment of myoblasts and cardiomyocytes in vitro results in improvements in mitochondrial function and alleviation of oxidative stress (Parker et al. 2018; Deng et al. 2021; Liu et al. 2020). More generally, DR has been shown to restore mitochondrial function and thereby ameliorate signs and symptoms of aging (Yu et al. 2020; López-Lluch et al. 2006; Zhou et al. 2021; Duszka et al. 2020; Hegab et al. 2019; Redman et al. 2018), part of which are mediated by upregulation of PPAR and PGC-1α (Hasan-Olive et al. 2019; Duszka et al. 2020). DR may also alleviate oxidative stress via upregulation of nuclear factor-erythroid 2-related factor (NRF2), a primary sensor of cellular stress and regulator of the expression of a range of enzymes with important detoxification and antioxidant functions (Lettieri-Barbato et al. 2020; Vasconcelos et al. 2019; Martin-Montalvo et al. 2011). Nonetheless, Xu et al. recently reported that long-term KD and βHB may in fact reduce mitochondrial biogenesis and increase cardiac fibrosis in human heart tissue (Xu et al. 2021), so further research is needed to untangle the effects of DR on mitochondrial function.

### Metabolic hormones

Age-related disorders are often associated with abnormal secretion and signaling of various metabolic hormones, including insulin and insulin-like growth factor (IGF-1) (Kolb et al. 2020; Ferreira 2021; Rose and Vona-Davis 2012). Insulin and IGF-1 act on glucose homeostasis by promoting cellular glucose uptake, regulate the carbohydrate, lipid, and protein metabolism (Clemmons 2012; Petersen and Shulman 2018), and enhance cellular proliferation via insulin/IGF1 receptor signaling (Hakuno and Takahashi 2018; Petersen and Shulman 2018). Numerous preclinical and clinical studies have identified a link between insulin secretion deficiency, insulin resistance, and hyper-insulinemia, which is a major cause of age-related diseases like cancer and diabetes (Bartke 2019; Shou, Chen, and Xiao 2020; Pak et al. 2021). DR lowers the secretion of both insulin and IGF-1 by limiting glucose intake and restraining the glucose metabolism (Urbain et al. 2017; Newman et al. 2017; Hopkins et al. 2018; Stubbs et al. 2018). DR can therefore improve insulin resistance (Cho et al. 2019; Forsythe et al. 2008; Sutton et al. 2018; Albosta and Bakke 2021). A recent study found while both CR and KD reduce blood glucose and insulin levels, only CR was able inhibit the growth of pancreatic tumor allografts in mice (Lien et al. 2021): the authors propose that a metabolic shift in the lipid metabolism and the reduced availability of lipids in the diet may drive the anti-tumor effects of CR. While KD also induced this metabolic shift, the increased availability of dietary fats abrogated the tumor growth-inhibiting effects. In contrast to the study by Lien et al., findings from other pancreatic mouse models showed that KD in combination with chemotherapy suppressed tumor growth by lowering insulin and glucose and increasing βHB levels (Yang et al. 2022). In addition to the latter study, several other studies also reported that KD can exert an anti-tumor effect even in the absence of an effect on plasma glucose levels (Weber et al. 2020). This raises the question of whether the composition of lipids in the KD may suppress tumor growth or whether other factors such as the type of animal model or tumor type could lead to different responses to the KD.

Ames (*Prop*^df^) and Snell (*Pit1(dw))* dwarf mice carry recessive mutations in pituitary genes resulting in a lack of growth hormone (GH) and have been found to exhibit an increased lifespan, indicating a disruption of the growth hormone axis could delay aging and/or promote longevity (Brown-Borg et al. 1996; Flurkey et al. 2001). Interestingly, the lifespan of Ames mice can be further extended by CR (Bartke et al. 2001), but supplementation of GH abrogates the beneficial effects of CR in both Ames and wild type mice (Gesing et al. 2014; Bonkowski et al. 2006). GH treatment during the early postnatal period in Ames mice can reduce lifespan, suggesting that early-life priming may result in metabolic or molecular memories that can, in part, explain developmental origins of aging phenotypes and disease (Sun et al. 2017).

The gut-related hormone ghrelin is another metabolic hormone involved in anti-aging and neuroprotection (Lips et al. 2014; Stoyanova 2014). IF or CR have been reported to elicit increased ghrelin secretion (Amitani et al. 2017; Bayliss and Andrews 2016; Al-Rawi et al. 2020; Amicis et al. 2019), although serum levels of ghrelin, melatonin, and leptin have been reported to decrease after diurnal IF (Al-Rawi et al. 2020). KD or ketone esters reduced ghrelin secretion in children with refractory epilepsy (Marchio, Roli, Lucchi, et al. 2019; Marchio, Roli, Giordano, et al. 2019) and healthy adults (Stubbs et al. 2018), but had no effect in children and adults with GLUT1-deficiency syndrome or refractory epilepsy (Amicis et al. 2019).

The ratio of the adipose tissue hormones adiponectin and leptin has been proposed as a biomarker of adipose tissue dysfunction. High leptin and low adiponectin plasma levels are associated with aging and obesity (Filippi and Lam 2014; Balasko et al. 2014; Goktas et al. 2005) as well as a poor prognosis for various malignancies such as breast, colon, and prostate cancer (Garofalo and Surmacz 2006; Artac and Altundag 2012). IF, ADF, and CR reduce circulating leptin (Balasko et al. 2014; Cho et al. 2019; Varady et al. 2013) and increase adiponectin levels (Varady et al. 2013; Cui et al. 2020; Wan et al. 2010; Varady et al. 2010), although some studies have found no effect on adiponectin levels (Varady et al. 2007; Gavrila et al. 2003; Rogozina et al. 2011). KD alongside regular exercise increased the ratio of adiponectin to leptin in adults (Cipryan et al. 2021). While long-term KD alone altered the levels of leptin in children and adolescents (Amicis et al. 2019), short-term KD increased the levels of adiponectin in obese adults (Monda et al. 2020).

The effects of DR on metabolic hormone levels appear to be strongly dependent on the type of DR, and further research will be required to pinpoint the exact roles hormones have in mediating the beneficial effects of DR.

### Cellular integrators of energy, nutrients, and growth factor signals: AMPK and mTOR

Various studies have shown that the AMPK–mTOR pathway is associated with longevity and senescence (Weichhart 2018). In response to bioenergetic stress, AMPK upregulates numerous catabolic pathways (e.g., fatty acid oxidation) to restore cellular ATP levels and modulates the activity of mTOR, an intracellular nutrient sensor that regulates protein synthesis, cell growth, metabolism, and inflammation (Weichhart 2018). Interestingly, Jordan et al. reported that AMPK activation not only coordinated the metabolic adaptation to fasting, but also regulated the pool of circulating inflammatory cells (Jordan et al. 2019).

During periods of nutrient deficiency, AMPK activation results in inhibition of mTOR (Xu, Ji, and Yan 2012). As DR induces a state of metabolic stress, it has been hypothesized that DR results in mTOR inhibition, but recent studies have painted a more complex picture, revealing that KD-induced downregulation of mTOR is independent of AMPK (Genzer et al. 2015). KD reduces the levels of essential amino acids (Aminzadeh-Gohari et al. 2017; Douris et al. 2015; Roberts et al. 2016; Weber et al. 2022), a process which may be involved in mTOR inhibition, in particular via downregulation of leucine and arginine (Weichhart 2018; Sheen et al. 2011). The effect of DR on the AMPK-mTOR pathway may vary by tissue. Murine studies showed that KD-induced bioenergetic stress activated AMPK in neuroblastoma or liver cells, reduced AMPK activation in retinal cells, and had no effect on muscle or brain cells (Aminzadeh-Gohari et al. 2017; Harun-Or-Rashid and Inman 2018; McDaniel et al. 2011). Similarly, mTOR activity in murine muscle tissue is increased by KD (Roberts et al. 2017; You et al. 2020), while it is decreased in the liver (Roberts et al. 2017; Genzer et al. 2015; McDaniel et al. 2011; Newman et al. 2017) and brain (Singh et al. 2018; Genzer et al. 2015; McDaniel et al. 2011).

### Sirtuins: NAD^+^-sensitive metabolic sensors

The sirtuin family (SIRT1–7) consists of evolutionarily conserved nicotinamide adenine dinucleotide (NAD^+^)-dependent lysine deacetylases involved in a variety of biological processes, including aging, cell survival and proliferation, apoptosis, DNA repair, and metabolism (Kratz et al. 2021). NAD^+^ is a substrate for all sirtuins. Reduced levels of both are observed in aging (Covarrubias et al. 2021; Akter et al. 2021). Several studies have indicated that the anti-aging effects of DR may be associated with an induction of sirtuins (Tang et al. 2021; Ma et al. 2020; Lilja et al. 2021; Palacios et al. 2009). Both in yeast and mammals, homologues of sirtuin SIRT2 mediate the lifespan-extending effects of CR (Imai et al. 2000; Mercken et al. 2014). Overexpression of *Sirt1* mimics CR and delays aging in mice (Satoh et al. 2013; Bordone et al. 2007). Deficiency of *SIRT6* leads to a shortened lifespan in mice and non-human primates, whereas SIRT6 overexpression and CR-induced SIRT6 activation both delay aging phenotypes (Kanfi et al. 2012). Similarly, a decrease in SIRT7 significantly attenuated the anti-tumor effects of IF (Tang et al. 2021).

Upregulation of NAD^+^ and sirtuins has been a topic of substantial interest for the prevention of age-related diseases (Braidy and Liu 2020; Elamin et al. 2017; Qin et al. 2006; Kane and Sinclair 2018; Akter et al. 2021; Yoshino et al. 2021). Following promising preclinical results, boosting NAD^+^ levels (and, subsequently, sirtuins) via supplementation has become a topical strategy to combat aging. Several clinical studies are currently ongoing to investigating safety and tolerability of NAD^+^ supplements, such as nicotinamide riboside (NR) or nicotinamide mononucleotide (NMN) (Reiten et al. 2021). DR may provide an alternative path to induce sirtuin levels: for example, *SIRT1* and *SIRT3* were found to be increased in blood after five days of periodic fasting in humans (Lilja et al. 2021).

### The immune system and inflammation

With advancing age, the immune system undergoes devitalizing changes, resulting in greater susceptibility to infection, inflammation, and autoimmunity (Ferrucci and Fabbri 2018; Muller, Benedetto, and Pawelec 2019). Individuals with age-related diseases have significantly higher serum levels of proinflammatory cytokines and chemokines. DR has been shown to reduce inflammation associated with inflammatory and autoimmune diseases without weakening the immune system against infections. For instance, KD increased the survival of COVID-infected mice by increasing tissue-protective T cells, reducing inflammation, and decreasing the number of pathogenic monocytes in the lungs (Ryu et al. 2021). Interestingly, like DR, treatment with fisetin, a naturally occurring senolytic compound that reduces senescent cell burden, has been shown to improve survival following SARS-CoV-2 challenge in old mice, indicating that removal of senescent cells could improve age-associated inflammation (Camell et al. 2021). Fisetin is now being explored for the prevention of COVID-19 complications in human clinical trials (NCT04771611, NCT04537299, and NCT04476953).

DR elicits immune-supportive responses and ameliorates inflammatory and autoimmune diseases, suggesting it may act in part as an immune adjuvant. DR stimulates lymphocyte-dependent killing of cancer cells (Buono and Longo 2019) and several in vitro and murine studies indicate that DR modulates the inflammatory response by reducing the levels of circulating pro-inflammatory cytokines (Forsythe et al. 2008; Moro et al. 2016; Faris et al. 2012; Goldberg et al. 2017; Youm et al. 2015; Lu et al. 2018; Monda et al. 2020; Harun-Or-Rashid and Inman 2018) and monocytes (Jordan et al. 2019). Furthermore, DR inhibits NOD-like receptor protein 3 (NLRP3)-inflammasome activation, an essential cytosolic regulator of innate immunity, in peripheral macrophages, neutrophils (Goldberg et al. 2017) and monocytes (Youm et al. 2015). Again, βHB appears to be a key mediator of the effects of DR on the immune system: βHB has been shown to reduce systemic inflammation via suppression of NLRP3-inflammasome formation in human monocytes (Youm et al. 2015), neutrophils (Goldberg et al. 2017) and cardiomyocytes (Youm et al. 2015; Byrne et al. 2020) as well as in rodent models of spinal cord injury, chronic unpredictable stress, and progressive eye abnormalities (Qian et al. 2017; Yamanashi et al. 2017; Harun-Or-Rashid and Inman 2018). Murine studies demonstrated that DR and βHB induce T cell-dependent anti-cancer effects, which result in cancer immunosurveillance and synergize with immune checkpoint blockade (Ferrere et al. 2021; Lussier et al. 2016).

### Epigenetic modulation by DR

While the exact mechanisms linking age, diet, DNA methylation and senescent cells are not yet clear, it is evident that DR increases lifespan, reduces the accumulation of senescent cells (Fontana, Nehme, and Demaria 2018; Messaoudi et al. 2006), and influences (age-related) methylation signatures (current literature summarized in (Ng et al. 2019)). Both DR and mTOR-interference, which influence similar pathways, delay age-related DNAme signatures or drift in mice and rats (Cole et al. 2017; Miyamura et al. 1993; Hadad et al. 2018; Kim et al. 2016; Hahn et al. 2017; Maegawa et al. 2017). CR in *Daphnia magna* leads to DNAme changes of genes involved in methylation, providing an epigenetic feed-forward mechanism (Hearn et al. 2019), and DR has been shown to epigenetically reprogram lipid metabolism in mice (Hahn et al. 2017). Recent studies provide insights into the mechanistic links between DR and epigenetic alterations: βHB acts as an endogenous histone deacetylase (HDAC) inhibitor (Shimazu et al. 2013) and modifies the expression of genes involved in DNAme, including sirtuins. Sirtuins modify DNAme to prevent premature activation of inflammatory genes in immune cells (Li et al. 2020), and regulate DNAme and differentiation potential in stem cells by antagonizing DNA methyltransferase (DNMT) (Heo et al. 2017). Sirtuins also influence DNAme at PCGT promoters (Wakeling et al. 2015; Furuyama et al. 2004), which are strongly implicated in aging and cancer (Widschwendter et al. 2007). Sirtuin expression therefore could provide a link between diet, the epigenome, and longevity/senescence, and ultimately health and disease.

Cellular metabolism and the epigenome are tightly linked (Finkel 2015), and the effects of DR interventions appear to result, at least in part, from the prevention or reversal of age-associated DNAme changes (Zhang et al. 2020). Glucose restriction in mice increases DNMT1 activity and triggers *p16INK4a* gene promoter hypermethylation, thereby reducing the expression of the senescence-associated protein p16INK4a (Li, Liu, and Tollefsbol 2010). Another strand of evidence for potential involvement of epigenetics in mediating the effects of DR comes from a recent study in transgenerational inheritance of longevity in *Caenorhabditis elegans*. Exposure of *C. elegans* to transient fasting (TF) influences mortality not only in the exposed generation (parental, P0), but also in at least four descendant generations (F1-F4) (Ivimey-Cook et al. 2021). Longevity can be inherited via epigenetic factors in *C. elegans*, which have been shown to exhibit a specialized type of DNAme (Greer et al. 2015; Greer et al. 2011). These data raise the possibility that epigenetic changes caused by DR could be inherited. Importantly, Ivimey-Cook et al. found that whereas TF reduced mortality in the P0 generation, it increased mortality in the F4 generation. While the mechanisms underlying this phenomenon have not yet been explored, the findings suggest a potential need to consider the health of offspring in the pursuit of longevity.

We may also gain insights into the molecular mechanisms contributing to DR-associated clearance of senescent cells and DNAme changes by observations of other ‘geroprotective’ therapeutics (i.e., therapeutics protecting from aging) that target similar pathways. For example, metformin, a widely prescribed antidiabetic drug, has been found to target several molecular mechanisms of aging and increase healthspan (Piskovatska et al. 2020), and epidemiological evidence suggests it can also reduce the incidence of cancers (Zhang et al. 2013; Kasznicki, Sliwinska, and Drzewoski 2014). Like DR (Lei and Lixian 2012; Weir et al. 2017), metformin treatment activates AMPK, and its antineoplastic effect may be mediated via modulation of the mTOR signaling pathway and DNAme (Pernicova and Korbonits 2014; Zhong et al. 2017; Yan et al. 2020). Metformin has been suggested to represent a metabolo-epigenetic regulator linking cellular metabolism to the DNA machinery (Cuyàs et al. 2018). Another geroprotective drug, rapamycin, which directly targets the mTOR pathway, extends lifespan in mice, even when animals are treated later in life (Harrison et al. 2009), and slows accumulation of epigenetic aging signatures in mouse hepatocytes similar to CR (Wang et al. 2017). Interestingly, however, two recent studies found distinct transcriptomic profiles between long-term CR and rapamycin treatment (Ham et al. 2022; Orenduff et al. 2022), as well as additive effects on counteracting muscle loss, potentially opening the option to parallel interventions counteracting age-related events.

In summary, DR may promote epigenetic rejuvenation and thus induce longevity and reduce cancer risk (Topart, Werner, and Arimondo 2020; Sen et al. 2016; Zhang et al. 2020). It is currently not clear whether DR reduces the proportion of aged and/or senescent cells, for instance by reduced formation of senescent cells, or a suppression of the senescent phenotype e.g. acting at an epigenetic level on individual cells to promote “epigenetic” rejuvenation. It is likely a combination of both: e.g., CR could trigger epigenetic rejuvenation of immune cells to accelerate clearance of senescent cells. An initial insight into the effects of CR and cellular aging on individual cells has been provided by Ma et al., who found that CR attenuates age-associated cell type-specific gene expression changes and relieves the accumulation of pro-inflammatory cells in various tissues (Ma et al. 2020).

## Not only what we eat, but when: merging roles of timing on the effects of DR

Recent studies dissecting the effects of calorie intake, fasting, and type of diet on DR have shed further light on what factors may mediate the beneficial effects on survival. In CR, the beneficial and anti-tumor effects have been proposed to be a result of reduced calorie intake, but a recent study by Pak et al. has overturned this long-held belief (Pak et al. 2021). CR in rodents is typically carried out using once-a-day feeding, resulting in fasting periods of up to 22 hours. The authors found that the same number of calories delivered by feeding three times a day rather than once (removing the effect of fasting) abrogated the beneficial effects of CR on metabolic health and longevity, suggesting fasting is required. Fasting alone also recapitulated many of the beneficial metabolic effects of CR (Pak et al. 2021). Timing of feeding and fasting also appears to be crucial. CR has been shown to be particularly successful in extending lifespan in mice when the animals fasted for at least 12 h during rest phases and consumed food during the active phase, “aligning” the feeding and fasting patterns to circadian rhythms (Acosta-Rodríguez et al. 2022). A recent study in humans investigating the effects of restricting feeding to 8 h either in the morning or the middle of the day found that only “early”, but not “mid-day”, time-restricted feeding produced beneficial effects on insulin sensitivity (Xie et al. 2022). In line with these findings, results from a 2-year CR study in humans (CALERIE trial) indicated that food consumption occurring earlier in the day (as opposed to later in the day) and smaller windows (i.e. longer fasting periods) were associated with bigger weight loss (Fleischer et al. 2022).

While both daily CR and a FMD regimen using caloric cycling (4 days severe CR, 10 days *ad libitum* feeding) reduced breast cancers in a mouse model, the protective effect was significantly higher in the daily CR group (Pomatto-Watson et al. 2021). A study in *Drosophila melanogaster* showed that distinct TRF schedules prolonged life and highlighted the need for further in-depth study of different DR mechanisms: the authors defined a life- and healthspan-extending schedule of TRF, but also identified that other schedules either reduced lifespan or did not alter lifespan at all (Ulgherait et al. 2021). Importantly, the lifespan-extending effects of TRF were dependent on an intact circadian clock, in line with other studies that suggest timing of feeding is crucial for the beneficial effects of DR. Thus, evidence from humans, mice, and *Drosophila* suggests that DR-induced anti-aging effects are linked to circadian rhythms (Singh et al. 2020; Pak et al. 2021; Jamshed et al. 2019).

## Limitations of current animal models and experimental approaches to study aging, and translation to human health

Many studies investigating aging have traditionally investigated lifespan or specific pathologies associated with age, yet it is important to distinguish the (albeit) fine line between age-associated specific pathologies or the general decline of physiological systems. For instance, neoplasms account for up to 90% of natural age-related deaths in laboratory mouse strains (Xie, Fuchs, et al. 2022) and hence “anti-aging” therapies that target neoplasms may therefore influence lifespan without influencing the aging process itself. Lifespan may therefore not always be a reliable proxy for aging. By comparing effect sizes of putative anti-aging interventions on a variety of biological parameters in both young and aged mice, a recent study indicated that many of these “anti-aging” interventions may in fact not target aging itself but other molecular pathways linked with pathology: many of the “anti-aging” effects of IF already manifested in young mice, at a time when no aging phenotypes were observed (Xie, Fuchs, et al. 2022). The authors concluded that IF may therefore target pathology-associated pathways rather than aging. These results indicate that stronger markers for aging and a dissection of underlying molecular and phenotypic changes is required for future studies.

Another limitation of many current studies investigating various DR is the common use of *ad libitum* feeding in controls. Compared to wild animals, *ad libitum* feeding in laboratory animals essentially constitutes a form of overfeeding, and hence is likely not comparable to a “normal feeding” state. It may overestimate the effects of DR on lifespan and pathology. Future studies including controls that more closely mimic normal feeding states are warranted (Feige-Diller et al. 2020).

Lastly, whether observations on changes in lifespan and disease following DR in various animal models can be translated to humans remains to be seen. In Table 4, we provide an overview of molecular findings from animal models that have – or have not – been confirmed in humans. As for longer-term and/or disease outcomes, a recent meta-analysis suggested that evidence for a beneficial effect of intermittent fasting is moderate to strong for weight loss but limited for other outcomes such as cardiovascular disease (Patikorn et al. 2021). Due to the much longer lifespan and many individual lifestyle factors of humans compared to laboratory animals that are kept in a tightly controlled environment, human studies investigating mortality outcomes are extremely challenging to virtually impossible, and only few studies so far have sufficient duration, participants, or compliance with the intervention to provide deep insights into key phenotypes such as cardiovascular, cancer, or mortality outcomes. Future studies investigating either disease outcomes or reliable “surrogate” markers of disease in humans are warranted.

## Conclusion and future questions

**Figure 1:**
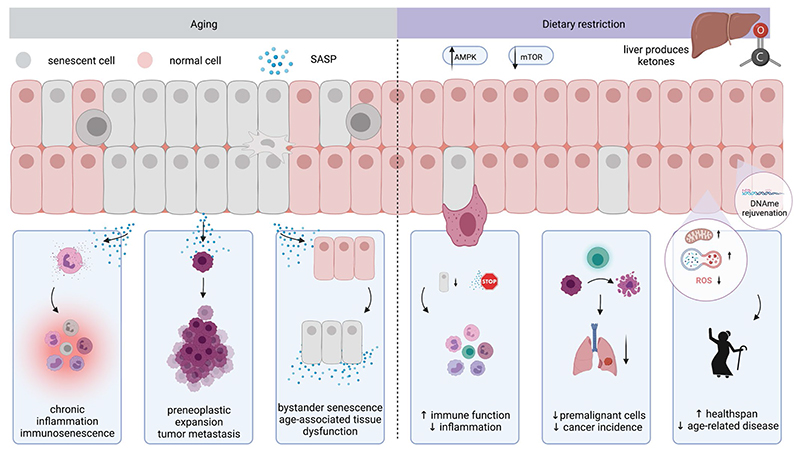
Schematic overview of involvement of senescent cells in aging and how DR may aid disease prevention or treatment and increase healthspan. Abbreviations: SASP, senescence-associated secretory phenotype; DNAme: DNA methylation; ROS: reactive oxygen species.

There is increasing evidence that DR reduces the accumulation of senescent cells and improves healthspan and lifespan. We propose that DR-associated senolysis may be achieved in part via metabolic reprogramming and epigenetic rejuvenation (summary and graphical overview in Figure 1). While we welcome the increasing interest in dietary, non-pharmacological approaches in longevity and prevention research, we emphasize the urgent requirement for further research to fully understand both the beneficial and potential harmful effects, including to future generations of offspring, and to further dissect the underlying molecular mechanisms. In particular, many preclinical studies to date have been carried out on male animals only (see Table 1), and more needs to be done to understand the effects of DR in females in relation to sex hormones and their fluctuation.

A hindrance in the interpretation of current studies is the lack of comparability between highly variable DR regimens and their effects in different species. More research is required to identify robust markers of the beneficial effects of DR, for instance on clearance of senescent cells, which could provide universal markers of DR efficacy. Circadian rhythms are likely to play a major role and timing of sampling may also influence results. Additionally, new research is required to understand the effects of DR on individual cells. Undoubtedly, future studies will assess senescence using single-cell technologies to gain a better understanding of the role of cellular heterogeneity and its relationship to dietary interventions. Cellular and molecular profiling of the effects of DR may enable the development of novel non-pharmacological strategies for longevity and disease prevention.

## Figures and Tables

**Table 1:** Diet and impact on lifespan and disease in selected preclinical studies.

Organism	Diet	Results	Molecular markers	Reference
Mouse (OF1, female)	ADF	↓ lymphoma incidence	↓ mitochondrial ROS ↑ splenic superoxide dismutase activity	(Descamps et al. 2005)
Mouse (C57BL/6J, male)	ADF	modulation of adipocyte function ↔ body and adipose tissue weight	↔ Adiponectin	(Varady et al. 2007)
Mouse (C57BL/6, DBA/J2, male & female)	Comparison of DR regimes	fasting is required for CR-induced metabolic benefits and lifespan extension	↑ insulin sensitivity ↑ fatty acid oxidation ↑ glucose sensitivity	(Pak et al. 2021)
Rhesus monkey (male & female)	CR	↓ aging-related deaths ↓ onset of age-associated pathologies: diabetes, cancer, cardiovascular disease & brain atrophy	↓ epigenetic age-associated drift	(Colman et al. 2009; Maegawa et al. 2017)
Rhesus monkey (male & female)	CR	↓ cancer incidence		(Mattison et al. 2017)
Mouse (C57BL/6J background)	CR	↓ aging-induced changes in lung ↑ epithelial cell survival in injured lung	↑ mitochondria & respiration ↓ inflammation	(Hegab et al. 2019)
Mouse (C56BL/6J, male)	CR with/without circadian alignment	↑ 30% or 35% increase in lifespan (without or with circadian alignment)	↓ age-related genes in liver	(Acosta-Rodríguez et al. 2022)
Mouse/rat – breast cancer model (MMTV-TGF-A/C57BL/6/Sprague-Dawley, female)	CR	↓ expression of proteins involved in the mTOR & IGF-I signaling pathways in mammary tissues	↑ pAMPK ↓ IGF-I ↓ mTOR	(Dogan et al. 2011), (Jiang, Zhu, and Thompson 2008; Dogan et al. 2011)
Rat (Sprague-Dawley, male)	CR	preservation of muscle mass in middle-aged rats but not younger rats	↓ mTOR	(Chen et al. 2019)
Mouse (TG2576, female)	CR	prevention of amyloid neuropathology	↑ Sirt1 expression ↑ NAD^+^	(Qin et al. 2006)
Mouse/rat – hypertension model (Wild type/Prkaa2^tm1.1Vio^ mutant; Sprague-Dawley; both male)	CR	↑ left ventricular function	↓ serum brain natriuretic protein ↑ mitochondrial biogenesis	(Niemann et al. 2022)
Mouse (C57BL6, male)	CR		↓ age-associated DNA methylation drift in hippocampus	(Hadad et al. 2018)
Mouse – breast cancer model (MMTV-TGF-A/C57BL/6, female)	CR (chronic or intermittent)	↓ tumor growth ↑ adiponectin in mammary fat pads in tumor-free mice in intermittent CR ↓ adiponectin signal in mammary fat pad in tumor-bearing mice	↓ leptin, ↔ adiponectin ↑ adiponectin/leptin ratio	(Rogozina et al. 2011)
Mouse – breast cancer model (BALB/CJ + 4T1, female)	CR/FMD	↓ primary breast cancer growth and metastasis	↓ CD11b^+^Gr1^+^ immune cells (tumor-promoting) ↑ CD4^+^ and CD8^+^ T cells (tumor-fighting)	(Pomatto-Watson et al. 2021)
Mouse – allogenic tumor graft model (C57BL/6J, male & female)	CR/KD	↓ cancer growth in CR but not KD	↓ stearoyl-CoA-desaturase ↓ lipid availability (CR)	(Lien et al. 2021)
*Caenorhabditis elegans*	DR (removal of food after larval stage)	↑ lifespan ↑ resistance to oxidative stress ↑ thermotolerance		(Lee et al. 2006)
*Saccharomyces cerevisiae*	FMD (switch nutrient-rich to water every 48 h, 3 cycles of PF during the lifespan)	↑ medium & maximum lifespan ↑ stress resistance to hydrogen peroxide		(Brandhorst et al. 2015)
Mouse (C57BL/6, female)	FMD (very low calorie/low protein for 4 days twice a month)	↓ cancer incidence, inflammation in tissues (lymph nodes, liver) ↓ immunosenescence ↑ hippocampal neurogenesis	↓ IGF-1 signaling	(Brandhorst et al. 2015)
Mouse (c57BL/6/BKS type 1 or Type 2 diabetes model, male)	FMD	↑ generation of insulin-producing β-cells	↑ Sox17, Ngn3	(Cheng et al. 2017)
*Drosophila melanogaster*	IF	↑ lifespan ↓ late-life gut pathology ↑ gut-barrier function		(Catterson et al. 2018)
Mouse – obese (C57BL/6J, BECN1^+/-^, LAMP2^-/-^, male & female)	IF	↑ beta cell survival ↑ glucose tolerance ↑ glucose-stimulated insulin secretion	↑ autophagy-lysosome pathway ↑ NEUROG3 (marker of pancreatic regeneration)	(Liu et al. 2017)
Mouse (tissue-specific ATG^-/-^, male)	IF (isocaloric twice-a-day feeding)	prevention of age/obesity-associated metabolic defects ↓ adiposity ↑ muscle mass.	↑ autophagy ↓ gluconeogenesis ↑ pAMPK	(Martinez-Lopez et al. 2017)
Rat – spinal cord injury (Sprague-Dawley, male)	IF	neuroprotection after acute spinal cord injury	↑ autophagy ↑ lysosomal function ↑ pAMPK	(Yuan et al. 2021)
Rat – myocardial infarction (Wistar, male)	IF	↑ glycemic control protection of myocardium against ischemia-induced cell damage & inflammation	↓ leukocyte infiltration ↓ plasma IL-6 ↑ adiponectin	(Wan et al. 2010)
Mouse – mammary and skin tumors (various transgenic mice, female)	IF	↑ anti-tumor effects in combination with chemotherapy & targeted therapy	↑ pAMPK ↑ SIRT7	(Tang et al. 2021)
Mouse (CD-1, male)	IF	↑ long-term memory and cortical thickness ↓ oxidative stress	↓ HDL, cholesterol,	Li, Wang, and Zuo 2013
Mouse (C57BL/6, male)	KD	↓ midlife mortality ↓ obesity ↑ memory ↔ maximum lifespan	↓ insulin, ↓ mTOR, protein synthesis pathways ↑ PPARα pathway in liver	(Newman et al. 2017)
Mouse (C57BL/6J background)	KD	↓ Colorectal cancer frequency via βHB	↑ HOXP ↓ epithelial proliferation, tumor growth	(Dmitrieva-Posocco et al. 2022)
Mouse (CD1-nu, female)	KD	↓ Neuroblastoma growth ↓ angiogenesis, while ↑ blood vessel maturation	↑ pAMPK ↑ ketosis ↓ glucose ↓ essential amino acids	(Aminzadeh-Gohari et al. 2017)
Mouse - COVID-19 model (C57BL/6, male)	KD	restraint of immune-dependent exacerbation of COVID	↑ tissue protective γδ T cells ↓ NLRP3 inflammasome ↓ pathogenic monocytes in lungs	(Ryu et al. 2021)
Mouse (C56BL/6J, male)	KD	↓ aging-associated myocardial remodeling & dysfunction	↑ autophagy ↓ oxidative stress ↓ ER stress	(Yu et al. 2020)
Rat – epilepsy model (Sprague-Dawley, male)	KD	↓ neuronal injury	↑ autophagy ↓ damaged mitochondria	(Wang et al. 2018)
Mouse (C57BL/6JN, male)	KD	prevention of age-related decrease in mitochondrial content	↑ mitochondrial citrate synthase ↑ complex I ↑ complex IV	(Zhou et al. 2021)
Mouse (mutUNG1/wild type, sex not known)	KD	↑ mitochondrial biogenesis & bioenergetics	↑ mitochondrial mass ↑ PGC1α ↑ SIRT3	(Hasan-Olive et al. 2019)
Mouse (C57BL/6, male)	KD	↑ median lifespan & survival	↑ acetylation ↓ mTORC1 signaling	(Roberts et al. 2017)
Rat (Sprague-Dawley, male)	KD	↑ brain health & increase of overall health via modulation of energy metabolism by acting on NAD+-dependent enzymes & their downstream pathways	↑ SIRT1 ↓ PARP-1 ↓ 8-hydroxy-2′-deoxyguanosine ↑ hippocampal NAD^+^/NADH ↓ DNA damage	(Elamin et al. 2018)
Mouse – glaucoma model (DBA/2J, male & female)	KD	anti-inflammation & neuroprotection in glaucoma	↓ TNFα ↓ IL-6 ↓ NOS2 ↓ pAMPK ↓ NLRP3 inflammasome	(Harun-Or-Rashid and Inman 2018)
Rat – Parkinson’s disease model (Wistar, male)	KD	↑ locomotor activity improvement normalization dopamine turnover in the striatum, KD may support late functional compensatory mechanisms for neurodegeneration		(Kuter et al. 2021)
Mouse – pulmonary fibrosis (male)	KD	↓ pulmonary fibrosis	↑ autophagy ↓ PI3K/AKT/mTOR signaling pathway	(Mu et al. 2021)
Mouse – Alzheimer’s model (APP C57BL x FVB, Female)	KD	↓ body weight ↔ cognitive function	↓ Aβ protein in brain	(Auwera et al. 2005)
Mouse – Alzheimer’s model (5XFAD C57Bl6, male)	KD	↑ spatial learning, working memory ↑ neurons in hippocampus and cortex	↓ Aβ protein in brain ↓ microglial activation	(Y. Xu et al. 2022)
*Caenorhabditis elegans*	TF	↓ mortality in exposed individuals, variable effects in F1-3 generations ↑ mortality in F4 generation		(Ivimey-Cook et al. 2021)
Mouse (C57BL/6J, male)	TF (24 h)	↑ antioxidant responses in skeletal muscle	↓ oxidative stress ↑expression of Nrf2-dependent genes	(Lettieri-Barbato et al. 2020)
Mouse (wild type C57BL6 or Fgf21^-/-^, male & female)	TF (24 h)		↓ oxidative stress response	(Kawakami et al. 2022)
Mouse – hypertension model (C56BL6, male & female)	TRF	↓ systolic blood pressure ↑ kidney function	↓ IL-6, IL-1 β	(Sims et al. 2022)
Mouse – chronic cerebral hypoperfusion model (C57BL6, male)	TRF	↑ learning ability ↓ neuropathology	alterations of DNA methylation	(Selvaraji et al. 2022)
*Drosophila melanogaster*	TRF	↑ life and healthspan with 6 h feeding, 20 h fasting starting mid-morning between days 10 and 40 ↓ or ↔ with other schedules (e.g. same feeding pattern in older flies, i.e. day 40-50; 24 h fasting followed by 1-2 days ad libitum feeding)	↑ circadian expression of autophagy mediators	(Ulgherait et al. 2021)

Various studies have shown beneficial effects of dietary restriction (DR) on health and lifespan in animal models. Abbreviations: ADF: alternate-day fasting; CR: caloric restriction; DIF: diurnal intermittent fasting; FMD: fasting-mimicking diet; GH: growth hormone; IF: intermittent fasting; KD: ketogenic diet; TF: transient fasting; TRF: time-restricted feeding. Signs: ↑: increase/improvement; ↓: decrease/attenuation; ↔: no effect.

**Table 2:** Diet and impact on lifespan and disease in selected human studies.

Population and type of trial	Diet / number of completed participants	Diet	Results	Molecular markers	Reference
Normal weight & overweight adults, RCT	CTRL = 15 ADF = 15	ADF	↓ weight ↓ fat mass cardio-protection	↑ adiponectin ↓ leptin	(Varady et al. 2013)
Healthy adults, RCT	CTRL = 19 CR = 34	CR	↑ resting energy efficiency	↓ ROS production	(Redman et al. 2018)
Adults aged 65+, observation	CR – Okinawa diet (epidemiological observation)		↑ lifespan ↓ risk for mortality from age-related diseases		(Willcox et al. 2007)
Healthy adults, RT	CTRL = 19 FMD = 19	FMD (3 months for 5 days FMD followed by 25 normal days)	↑ mesenchymal stem & progenitor cells in peripheral blood	↓ CRP ↓ IGF-1	(Brandhorst et al. 2015)
Healthy men, BCS	FMD = 8	FMD	↑ phenylalanine release in skeletal muscle	↑ net phenylalanine release ↓ circulating levels of insulin ↓ mTOR ↑ autophagy	(Vendelbo et al. 2014)
Healthy adults, RCT	CTRL = 16 FMD = 34	FMD (low sugar, calorie, protein, high in unsaturated fat)	↓ weight, trunk and total body fat mass ↓ blood pressure	↓ IGF-1	(Wei et al. 2017)
Healthy adults, BCS	IF = 14	IF	↓ weight, BMI	↑ key regulatory proteins in DNA repair ↑ proteins protective from cancer and diabetes	(Mindikoglu et al. 2020)
Men with prediabetes, RT	CTRL = 8 TRF = 8	early TRF	↑ cardiometabolic health	↑ insulin sensitivity ↑ β cell function ↓ blood pressure ↓ oxidative stress ↔ inflammation	(Sutton et al. 2018)
Overweight & obese adults	IF = 57	IF	gender & anthropometric variables impacted on the tested hormones	↓ ghrelin ↓ melatonin ↓ leptin ↔ cortisol	(Al-Rawi et al. 2020)
Women with breast cancer, NR & BCS	CTRL = 30 KD = 29	KD	↑ quality of life ↓ increase in symptom severity	↓ free T3	(Klement, Weigel, and Sweeney 2021)
Male athletes, RCT	CTRL = 10 KD = 9	KD	↓ fat mass	↓ glucose ↓ circulating insulin ↓ IL-6, TNFα	(Paoli et al. 2021)
Children with pharmacoresistant epilepsy, BCS	KD = 6	KD	↔ BMI	↓ ghrelin & des-acyl ghrelin	(Marchio, Roli, Giordano, et al. 2019; Marchio, Roli, Lucchi, et al. 2019)
Patients with glut1-deficiency syndrome & refectory epilepsy, BCS	KD = 30	KD	↔ BMI ↔ fat mass and lipid profiles	↔ ghrelin & leptin ↔ blood glucose, ↓ fasting insulin	(Amicis et al. 2019)
Overweight adults with atherogenic dyslipidemia, RCT & BCS	LFD = 20 VLCKD = 20	KD	↓ weight ↑ glycemic control & insulin sensitivity ↓ inflammation	↓ TNFα, IL-6, IL-8, MCP-1	(Forsythe et al. 2008)
Alzheimer’s disease patients, BCS	N = 10	KD	↑ Alzheimer’s Disease Assessment Scale-cognitive subscale (reverted to baseline after washout period)		(Taylor et al. 2018)
Alzheimer’s disease patients, RCT/X	N = 21	KD	↑ quality of life and daily activity scores ↓ weight, BMI	↓ HbA1c ↓ HDL, LDL, total cholesterol	(Phillips et al. 2021)
Elderly non-demented adults, BCS	N = 19	KD	↑ cognitive function (digit span test, Trail-Making Test B, and the global score)		(Ota et al. 2016)
Obese adults, BCS	VLCKD = 20	KD (very low calorie)		↑ adiponectin ↑ IL-10 ↓ insulin ↓ TNFα, CRP	(Monda et al. 2020)
Healthy adults, CC	CTRL = 12 KD = 21	KD (very low calorie)		alterations in DNA methylation	(Crujeiras et al. 2021)
Adults with epilepsy, BCS	N = 58	KD		alterations in DNA methylation (global loss)	(Pedersen et al. 2022)
Healthy adults, BCS	CTRL + regular exercise = 12 KD + regular exercise = 12	KD + regular exercise	↓ weight, fat mass	↑ adiponectin ↓ leptin	(Cipryan et al. 2021)
Adults aged 35-70 from 5 continents and 18 countries, prospective observational cohort	N = 135335	“KD-like” (observation of dietary carbohydrate and fat intake)	↑ mortality and cardiovascular disease with increasing carbohydrate intake (in particular >80% of energy intake from carbohydrates) ↓ mortality and cardiovascular disease with increasing total fat intake		(Dehghan et al. 2017)
Healthy adults, BCS	CTRL = 31 PF = 20	PF	↓ weight	↑ ketosis ↑ sirtuin expression ↑ *Christensenella spp*.	(Lilja et al. 2021)
Women without a known history of diabetes, BCS	TF = 121	TF (48 h)		↓ leptin ↔ adiponectin	(Gavrila et al. 2003)
Overweight adults, RCT/X	N = 11	TRF	changes in circadian gene expression, anti-aging, and autophagy markers	in the morning: ↑ ketosis, ↑ SIRT1, ↑ LC3A and ATG12 expression in blood in the evening: ↑ brain-derived neurotropic factor & mTOR expression	(Jamshed et al. 2019)
Healthy adults, RCT	CTRL = 28 Early-day TRF = 28 mid-day TRF = 26	TRF (eating for no longer than 8 h during 06:00-15:00 or 11:00-20:00)	↓ weight, fat mass ↓ HOMA-IR	↑ insulin sensitivity ↓ TNFα, IL-1β ↑ microbial diversity	(Xie et al. 2022)
Healthy adults, RCT (CALERIE study)	CTRL = 71 CR = 117 (main report)	25% CR (true CR achieved: 12% [mean])	↓ weight (bigger weight loss with food consumption earlier in the day and smaller eating windows) ↓ systolic and diastolic blood pressure ↑ thymopoiesis ↑ liver function (greater in men)	↓ LDL and cholesterol to HDL ratio, metabolic syndrome score ↓ CRP ↑ insulin sensitivity ≈ epigenetic age markers in blood samples	(Kraus et al. 2019; Spadaro et al. 2022; Fleischer et al. 2022; Dorling et al. 2021; Waziry et al. 2021; Ramaker et al. 2022)
Healthy adults, BCS	N = 5	FMD		↓ IGF-1	(Cheng et al. 2017)

Abbreviations: ADF: alternate-day fasting; BCS: baseline-controlled study; CC, case-control study (intervention versus control); CTRL: control; CR: caloric restriction; LFD: low fat diet; FMD: fasting-mimicking diet; IF: intermittent fasting; KD: ketogenic diet; NR: non-randomized; PF: periodic fasting; RCT: randomized controlled trial; RT: randomized trial; RCT/X: randomized controlled trial with crossover; VLCKD: very low carbohydrate ketogenic diet. Signs: ↑: increase/improvement; ↓: decrease/attenuation; ↔: no effect.

**Table 3:** Selected studies on elimination of pathological effects caused by senescent cells, alleviating age-related diseases.

Organism	Pathology	Strategy to target senescent cells	Effect of senolysis	Reference
Mouse	Chronic kidney disease	ABT-263 (Navitoclax)	↑ renal function and repair	(Mylonas et al. 2021)
Mouse	Cigarette smoke-induced emphysema	CDKN2A knockout (p16^-/-^)	↓ emphysema by promoting IGF1/Akt1 signaling	(Cottage et al. 2019)
Mouse	COVID-19 infection	Fisetin or INK-ATTAC mouse model	↓ inflammation and mortality after pathogen challenge	(Camell et al. 2021)
Mouse	Progeria	ABT-737	↑ median survival	(Ovadya et al. 2018)
Mouse	Normal aging	SSK1 (Senescence specific killing prodrug 1)	↓ inflammation, restoration of physical function	(Cai et al. 2020)
Mouse	Transplanted senescent cells	dasatinib and quercetin (D + Q)	↓ physical dysfunction, mortality hazard by 65%	(Xu et al. 2018)
Mouse	Normal aging	dasatinib and quercetin (D + Q)	↑ cardiac function and carotid vascular reactivity, lifespan	(Zhu et al. 2015)
Mouse	Chronic liver disease model	dasatinib and quercetin (D + Q)	↓ incidence of hepatocellular carcinoma ↓ senescence, senescence-associated secretory phenotype ↓ necroptosis	(Thadathil et al. 2022)
Mouse	Normal aging	Fisetin	↑ median and maximum lifespan, ↓ age-related pathology	(Yousefzadeh et al. 2018)
Mouse	Normal aging	FOXO4-p53 interfering peptide	↑ fitness, hair density and renal function	(Baar et al. 2017)
Mouse	Insulin resistance	INK-ATTAC mouse model or ABT-263 (Navitoclax)	↑ glucose metabolism and restoration of gene expression	(Aguayo-Mazzucato et al. 2019)
Mouse	Parkinson’s disease (environmental toxin model)	p16-3MR mouse model + ganciclovir	protection against toxin-induced neuropathology	(Chinta et al. 2018)
Mouse	Atherosclerosis	p16-3MR mouse model + ganciclovir	↓ streak size, ↓ expression of inflammatory markers, stabilization of fibrous caps	(Childs et al. 2016)
Mouse	Osteoarthritis	p16-3MR mouse model + ganciclovir	↓ post-traumatic osteoarthritis, ↑ cartilage development, reduced pain	(Jeon et al. 2017)
Mouse	Osteoporosis	INK-ATTAC mouse model or dasatinib and quercetin (D + Q)	↑ bone mass and strength and better bone architecture in aged mice	(Farr et al. 2017)
Mouse	Tau-dependent neurodegeneration (Alzheimer’s disease model)	INK-ATTAC mouse model	preservation of cognitive function	(Bussian et al. 2018)
Mouse (obese)	Metabolic dysfunction	INK-ATTAC mouse model	alleviation of metabolic and adipose tissue dysfunction	(Palmer et al. 2019)
Mouse (obese)	Susceptibility to ischemic injury due to obesity-induced vascular senescence	rapamycin treatment	↓ senescence by rapamycin treatment, ↔ body weight, prevention of limb necrosis and ischemic stroke	(Wang et al. 2009)
Mouse (obese)	Anxiety	INK-ATTAC mouse model or dasatinib and quercetin (D + Q)	↓ anxiety and lipid accumulation, restoration of neurogenesis	(Ogrodnik et al. 2019)
Mouse (obese)	Obesity-induced hepatocellular carcinoma (via SASP)	*Hsp47* siRNA liposomes	Prevention of hepatocellular carcinoma development	(Yoshimoto et al. 2013)

Signs: ↑: increase/improvement; ↓: decrease/attenuation; ↔: no effect.

**Table 4:** Comparison of animal models and human studies on the effects of DR or senolysis.

Effect ∕ finding	Animal studies and interventions	Reproduced in humans	Details	Population	Intervention	Citation
Alteration of metabolic hormones						
↓ *circulating IGF-1, insulin, or ↑ insulin sensitivity*	**CR** – mouse, breast cancer model (Jiang, Zhu, and Thompson 2008; Dogan et al. 2011); **FMD** – mouse, aged female (Brandhorst et al. 2015); mouse, diabetes model (Cheng et al. 2017); **IF** – mouse, obese (Liu et al. 2017); rat, myocardial infarct model (Wan et al. 2010); **KD** – mouse, male (Newman et al. 2017)	Yes	↓ IGF-1	Healthy adults, RT, n = 16	FMD	(Wei et al. 2017)
		Yes	↓ IGF-1	Healthy adults, BCS, n = 5	FMD	(Cheng et al. 2017)
		Yes	↓ IGF-1	Healthy adults, RT, n = 19	FMD	(Brandhorst et al. 2015)
		Yes	↓ circulating insulin	Healthy men, BCS, n = 8	FMD	(Vendelbo et al. 2014)
		Yes	↓ circulating insulin	Male athletes, RCT, n = 19	KD	(Paoli et al. 2021)
		Yes	↓ fasting insulin	Patients with glut1-def syndrome and refractory epilepsy, BCS, n = 30	KD	(Amicis et al. 2019)
		Yes	↓ circulating insulin	Obese adults, BCS, n = 20	VLCKD	(Monda et al. 2020)
		Yes	↑ insulin sensitivity	Healthy adults, RCT, n = 82	TRF (early versus late)	(Xie et al. 2022)
		Yes	↑ insulin sensitivity	Healthy adults, RCT, n = 188	CR	(Kraus et al. 2019)
Inflammation or inflammatory markers						
↓ *inflammation or inflammatory markers* e.g. ↑ γô T cells, ↓ NLRP3 inflammasome	**CR** – mouse (Hegab et al. 2019); mouse, breast cancer model (Pomatto-Watson et al. 2021); **FMD** – mouse, female (Brandhorst et al. 2015); **IF** – rat, myocardial infarct model (Wan et al. 2010); **KD** – mouse, COVID-19 model (Ryu et al. 2021); **TF** – mouse, male (Lettieri-Barbato et al. 2020); **TRF** – mouse, hypertension model (Sims et al. 2022)	Yes	↓ CRP	Healthy adults, RCT, n = 38	FMD	(Brandhorst et al. 2015)
		No	↔ inflammation	Men with prediabetes, RT, n = 16	IF	(Sutton et al. 2018)
		Yes	↓ IL-6, TNFα	Male athletets, RCT, n = 19	KD	(Paoli et al. 2021)
		Yes	↓ TNFα, IL-6, IL-8, MCP-1	Overweight adults with atherogenic dyslipidemia, RCT/BCS, n = 40	KD/VLCKD	(Forsythe et al. 2008)
		Yes	↓ TNFα, CRP; ↑ IL-10	Obese adults, BCS, n = 20	VLCKD	(Monda et al. 2020)
		Yes	↓ TNFα, IL-1β	Healthy adults, RCT, n = 82	TRF (early versus late)	(Z. Xie, Fuchs, et al. 2022)
		Yes	↓ CRP	Healthy adults, RCT, n = 188	CR	(Kraus et al. 2019)
Oxidative stress						
↓ *oxidative stress* e.g. ↓ mitochondrial ROS production, ↑ resistance to oxidative stress	**ADF** – mouse, age-associated lymphoma model (Descamps et al. 2005); **DR** – *Caenorhabditis elegans* (Lee et al. 2006); TF – mouse (Li, Wang, and Zuo 2013); mouse, wild type and Fgf21-/-^#^ (Kawakami et al. 2022); **KD** – mouse (Yu et al. 2020) ^#^ 24h one-off fast	Yes	↓ 8-isoprostane	Men with prediabetes, RT, n = 16	TRF (early)	(Sutton et al. 2018)
		Yes	↓ ROS production	Healthy adults, RCT, n = 53	CR	(Redman et al. 2018)
Autophagy						
↑ *autophagy markers*	**IF** – mouse, obese (Liu et al. 2017); mouse (Martinez-Lopez et al. 2017); rat, spinal cord injury model (Yuan et al. 2021); **KD** – mouse (Yu et al. 2020); rat, epilepsy model (Wang et al. 2018); mouse, glaucoma model (Harun-Or-Rashid and Inman 2018); mouse, pulmonary fibrosis (Mu et al. 2021)	Yes	↑ LC3B-II (autophagy marker) in muscle	Healthy men, BCS, n = 8	FMD	(Vendelbo et al. 2014)
		Yes	↑ *LC3A* and *ATG12* expression in blood	Overweight adults, RCT, n = 11	TRF	(Jamshed et al. 2019)
Epigenetic DNA methylation changes						
↓ *epigenetic age*	**CR** – Rhesus monkey (Maegawa et al. 2017), mouse (Hadad et al. 2018)	?	↓ DunedinPACE of aging, ↔ other epigenetic age markers	Healthy adults, RCT, n = 197	CR	(Waziry et al. 2021)
		?	DNA methylation altered, epigenetic age not directly studied	Healthy adults, intervention versus normal controls, CC, n = 33	KD	(Crujeiras et al. 2021)
		?	DNA methylation altered (global loss), epigenetic age not directly studied	Adults with epilepsy, BCS, n = 58	KD	(Pedersen et al. 2022)
		Yes	↓ epigenetic age acceleration	Healthy adults aged 65-79, RCT, n = 120	Mediterranean diet (described by Berendsen et al. 2014)	(Gensous et al. 2020)
Clinical change						
↓ *weight, fat mass, BMI*	**IF** – mouse (Martinez-Lopez et al. 2017); **KD** – mouse, male (Newman et al. 2017); mouse (Auwera et al. 2005)^§^ ^§^studies non-exhaustive, others have also demonstrated weight loss.	Yes	↓ weight, fat mass	Normal/overweight adults, RCT, n = 30	ADF	(Varady et al. 2013)
		Yes	↓ weight, trunk and total fat mass	Healthy adults, RT, n = 16	FMD	(Wei et al. 2017)
		Yes	↓ weight, BMI	Healthy adults, BCS, n = 14	IF	(Mindikoglu et al. 2020)
		Yes	↓ fat mass	Male athelets, RCT, n = 19	KD	(Paoli et al. 2021)
		No	↔ BMI	Children with pharmacoresistant epilepsy, BCS, n = 6	KD	(Marchio, Roll, Giordano, et al. 2019; Marchio, Roll, Lucchi, et al. 2019)
		No	↔ BMI, fat mass	Patients with glut1-def syndrome and refractory epilepsy, BCS, n = 30	KD	(Amicis et al. 2019)
		Yes	↓ weight	Overweight adults with atherogenic dyslipidemia, RCT/BCS, n = 40	KD/VLCKD	(Forsythe et al. 2008)
		Yes	↓ weight, fat mass	Healthy adults, BCS, n = 24	KD plus exercise	(Cipryan et al. 2021)
		Yes	↓ weight	Healthy adults, BCS, n = 51	PF	(Lilja et al. 2021)
		Yes	↓ weight, fat mass	Healthy adults, RCT, n = 82	TRF (early versus late)	(Xie et al. 2022)
		Yes	↓ weight	Healthy adults, RCT, n = 185	CR	(Kraus et al. 2019)
		Yes	↓ weight, BMI	Alzheimer’s patients, RCT/X, n = 21	KD	(Phillips et al. 2021)
↑ *cardiometabolic health* e.g. ↓ blood pressure, ↑ cardiac vascularity	**CR** – mouse, rat (Niemann et al. 2022); **FMD** – mouse, female (Mishra et al. 2021); **TF** – mouse, wild type and Fgf21-/-^#^ (Kawakami et al. 2022); **TRF** – mouse, hypertension models (Sims et al. 2022) ^#^ 24h one-off fast	Yes	↓ blood pressure	Healthy adults, RT, n = 16	FMD	(Wei et al. 2017)
		Yes	↓ systolic and diastolic blood pressure, ↔ arterial stiffness	Men with prediabetes, RT, n = 16	TRF (early)	(Sutton et al. 2018)
		Yes	↓ systolic and diastolic blood pressure	Healthy adults, RCT, n = 185	CR	(Kraus et al. 2019)
Aging and aging-associated pathology						
↓ *neurodegeneration* e.g. ↑ cognitive function, ↓ amyloid pathology	**CR** – mouse, female (Qin et al. 2006); **FMD** – mouse, female (Brandhorst et al. 2015); **IF** – mouse (Li, Wang, and Zuo 2013); **KD** – mouse, Alzheimer’s model (Auwera et al. 2005; Xu et al. 2022); rat, Parkinson’s model, male (Kuter et al. 2021); **TRF** – mouse, chronic cerebral hypoperfusion model, male (Selvaraji et al. 2022)	Yes	↑ cognitive subscale	Alzheimer’s disease patients, BCS, n = 10	KD	(Taylor et al. 2018)
		Yes	↑ scale improvement for daily living activities and Addenbrookes Cognitive Examination	Alzheimer’s disease patients, RCT/X, n = 21	KD	(Phillips et al. 2021)
		Yes	↑ cognitive function (digit span test, Trail-Making Test B, and the global score)	Elderly non-demented individuals, BCT, n = 19	KD	(Ota et al. 2016)
↓ *senolytic cell burden*	Senolytic treatment with dasatinib & quercetin (Thadathil et al. 2022)	Yes	↓ senescent cell burden in adipose tissue and skin ↓ IL-lα, IL-6, MMP-9 and -12	Patients with diabetic kidney disease, BCS, n = 9	Senolytic (Dasatinib + Quercetin)	(Hickson et al. 2019)
↑ lifespan, ↓ non-neurodenegerative age-related disease and cancer*	**ADF** – mouse, age-associated lymphoma model (Descamps et al. 2005); **CR** – Rhesus monkey, aging-related death (Colman et al. 2009); Rhesus monkey, cancer incidence (Mattison et al. 2017); mouse (Pak et al. 2021); mouse, male (Acosta-Rodríguez et al. 2022); mouse, breast cancer model; (Rogozina et al. 2011); *Drosophila melanogaster* (Catterson et al. 2018); **DR** – *Caenorhabditis elegans* (Lee et al. 2006); **FMD** – mouse, female (Brandhorst et al. 2015); **KD** – mouse, male (Roberts et al. 2017); mouse (Dmitrieva-Posocco et al. 2022; Yu et al. 2020); **TRF** – *Drosophila melanogaster* (Ulgherait et al. 2021)	Yes / ?	↑ lifespan	Epidemiological observation, adults aged 65+	CR (Okinawa diet)	(Willcox et al. 2007)
		Yes / ?	↑ mortality and cardiovascular disease with increasing carbohydrate intake ↓ mortality and cardiovascular disease with increasing total fat intake	Prospective observational cohort study, n = 135,355	Observation of dietary intake of carbohydrates and fat (KD-like diets)	(Dehghan et al. 2017)

* No major RCT studies with dietary intervention and mortality or disease outcome in humans so far. This is likely not feasible (require large n, long follow-up, long-term compliance required), or at least difficult to achieve in practice, and may be challenging ethically.

Overview of molecular, metabolic, cellular, clinical, and longevity/disease findings that could be reproduced (or not) between animal models and human studies so far. Abbreviations: n/a, not applicable; n/s, not studied; ADF: alternate-day fasting; BCS: baseline-controlled study; CC, case-control study (intervention versus control); CTRL: control; CR: caloric restriction; LFD: low fat diet; FMD: fasting-mimicking diet; IF: intermittent fasting; KD: ketogenic diet; NR: non-randomized; PF: periodic fasting; RCT: randomized controlled trial; RT: randomized trial; RCT/X: randomized controlled trial with crossover; VLCKD: very low carbohydrate ketogenic diet. Signs: ↑: increase/improvement; ↓: decrease/attenuation; ↔: no effect.

## Box 1 Types of Dietary Restriction (DR)

**Caloric restriction (CR)**: Limiting caloric intake to a certain amount per day without malnutrition

**Intermittent fasting (IF)**: Limitation of energy intake to certain periods, such as 5:2 intermittent fasting (food intake for 5 days, fasting for 2 days), but also including other intermittent fasting schedules such as:

- **Time-restricted feeding (TRF):** Food consumption limited to certain periods of the day, e.g. 16:8 (feeding for 8 h a day), or 18:6 (feeding 6 h a day, fasting for the remaining 18). Variations of this can define the time during which food is consumed, including **eTRF** (early TRF, feeding restricted to a period during early hours of the day).
- **Alternate day fasting (ADF):** Fasting and normal food consumption in alternating day patterns

**Ketogenic diet (KD):** High-fat, low-carb (or very low-carb) diet designed to simulate the state of fasting while supplying nutrients and calories; also sometimes referred to as **fasting-mimicking diet (FMD)**. KD and other FMDs constitute special meal plans that do not involve fasting, but instead aim to elicit similar metabolic benefits by restricting access to certain food components.

## Box 2 The Concept of Senolysis

Composite of the words *senescere*, to grow old, and *lytic*, to loosen, unfasten, or destroy, the term **senolysis** was coined to describe the process of selective elimination of senescent and aged cells. In recent years, senolysis has become a central focus of aging research. It is hypothesized that removal of senescent cells may aid tissue function, delay age-related symptoms, and even increase the healthspan by removing nonfunctional or actively harmful (e.g., via release of cytokines) cells. A variety of studies are ongoing to identify successful senolytic strategies, including small molecules such as dasatinib and quercetin (Kirkland and Tchkonia 2020; Robbins et al. 2021; Zhu et al. 2015; Chaib, Tchkonia, and Kirkland 2022), and lifestyle changes, such as described in this review.
